# Brain and Muscle Metabolic Changes by FDG-PET in Stiff Person Syndrome Spectrum Disorders

**DOI:** 10.3389/fneur.2021.692240

**Published:** 2021-09-17

**Authors:** Yujie Wang, Mohammad S. Sadaghiani, Fan Tian, Kathryn C. Fitzgerald, Lilja Solnes, Scott D. Newsome

**Affiliations:** ^1^Department of Neurology, Johns Hopkins University School of Medicine, Baltimore, MD, United States; ^2^Department of Radiology, Johns Hopkins University School of Medicine, Baltimore, MD, United States

**Keywords:** stiff person spectrum disorders, cerebellar ataxia, FDG–PET, anti-GAD 65 antibody, autoimmune disease

## Abstract

**Objective:** To report clinical characteristics and fluorodeoxyglucose positron emission tomography (FDG-PET) findings in the brain and muscles of individuals with stiff person syndrome (SPS) spectrum disorders (SPSSDs).

**Methods:** Retrospective cohort study from 1997 to 2018 at Johns Hopkins Hospital identified 170 individuals with SPS or cerebellar ataxia (CA) associated with anti-glutamic acid decarboxylase (anti-GAD)-65 antibodies. Fifty-one underwent FDG-PET, with 50 involving the body and 30 with dedicated brain acquisition. The clinical and immunological profiles were extracted via medical record review. The brain scans were analyzed quantitatively using the NeuroQ software, with comparison with an averaged normal database. The body scans were reviewed qualitatively by a blinded nuclear medicine radiologist.

**Results:** Mean age of symptom onset was 41.5 years (range 12–75 years). Majority were female (68%) and White (64%). Of the patients, 82% had SPS (majority being classic phenotype), and 18% had CA. Three had a paraneoplastic process. Forty-seven had serum anti-GAD, two with anti-amphiphysin, and one with anti-glycine receptor antibodies. Brain metabolic abnormalities were seen in both SPS and CA, with significant differences between the groups noted in the right superior frontal cortex, right sensorimotor cortex, left inferior parietal cortex, bilateral thalami, vermis, and left cerebellum. Of the patients, 62% demonstrated muscle hypermetabolism, most commonly bilateral, involving the upper extremities or axial muscles. Neither brain nor muscle metabolism was correlated with functional outcomes nor treatments.

**Conclusions:** Metabolic changes as seen by FDG-PET are present in the brain and muscle in many individuals with SPSSD. Future studies are needed to assess whether FDG-PET can help aid in the diagnosis and/or monitoring of individuals with SPSSD.

## Introduction

Anti-glutamic acid decarboxylase-65 (anti-GAD65) autoantibodies have been identified in a variety of rare neurologic disorders; (1–3) most frequently in stiff person syndrome (SPS), a condition characterized by muscle rigidity and overlying painful spasms, typically affecting the axial and limb musculature (3, 4). Other distinct phenotypes include cerebellar ataxia (CA), (2, 5) progressive encephalomyelitis with rigidity and myoclonus (PERM), (6, 7) epilepsy, (8) and encephalitis (9). There may be overlapping signs and symptoms in these phenotypes, (5, 10, 11) which point toward a spectrum of presentations. Besides anti-GAD65, other less common autoantibodies can be seen with SPS spectrum disorders (SPSSDs) including anti-amphiphysin (12) and anti-glycine receptor antibodies (6, 13). There is a suspected association with the impairment of γ-aminobutyric acid (GABA) neurotransmission by the aforementioned autoantibodies (with several locations implicated based on prior studies, such as the sensorimotor cortex), which results in a lack of inhibition within the nervous system leading to a relatively hyper-excitable state (4, 14). Diagnosis can be challenging, given their rarity and varied presentations (3, 13, 15). Anti-GAD65 antibodies are also absent in some individuals with the disease (3, 13) and can be seen in non-neurologic conditions, most commonly diabetes mellitus and thyroid disease, (3) though generally, the antibodies are present at a lower titer in non-neurologic disease. For SPS, various diagnostic criteria have been proposed, (16, 17) though diagnosis requires a high index of suspicion and rely on clinical presentation. The presence of an autoantibody is helpful in aiding in the diagnosis, especially if of high-titer, (10) though it has not been shown to correlate with disease burden or prognosis. (18) In general, imaging studies such as magnetic resonance imaging (MRI) are unhelpful in the diagnosis (besides ruling out alternative conditions), (13) and there are limited reports of the use of other imaging modalities, such as MR spectroscopy to measure GABA levels in the brain (14) and 2-deoxy-2-[fluorine-18]fluoro-d-glucose integrated with computed tomography (FDG-PET). FDG-PET has been increasingly recognized to provide valuable information in neuroimmunological conditions, such as autoimmune encephalitis (19–22). We sought to evaluate FDG-PET abnormalities present in the brain and muscles of individuals with SPSSD.

## Materials and Methods

### Standard Protocol Approvals, Registrations, and Patient Consents

The Johns Hopkins Institutional Review Board approved the study. All participants provided written informed consent as part of an ongoing, longitudinal observational study at the Johns Hopkins SPS Center.

### Design, Study Population, and Inclusion/Exclusion Criteria

This study is a retrospective cohort study of individuals seen at Johns Hopkins Hospital from January 1, 1997, to June 1, 2018, with the diagnosis of SPS with presence of an associated auto-antibody (anti-GAD65, anti-amphiphysin, or anti-glycine receptor) or anti-GAD65 related CA and who had an available FDG-PET study. The autoantibodies were detected in serum via commercially available methods at the time of testing. A total of 170 individuals were identified, out of which 51 had an available FDG-PET study. The standard clinical practice at the center is to obtain FDG-PET scans to screen for malignancy if neurologic symptom onset was within the previous 5 years, based on previous studies (23). Out of these, a total of 50 had body FDG-PET (one body scan was not available), and 30 had dedicated brain FDG-PET scans. If the individual had more than one FDG-PET scan, the first study was used. The main exclusion criteria included having a low-quality FDG-PET study or the presence of refractory epilepsy and/or encephalitis (including limbic encephalitis and PERM). The median time from symptom onset to acquisition of FDG-PET scan was 58 months (range 0–333 months).

### Clinical Phenotype and Disability Measure

For SPS phenotype, we further divided into classic SPS and SPS-plus, with classic SPS defined as individuals with lower extremity and/or lumbar stiffness and spasms, and SPS-plus if some of the classic symptoms/signs were present and if other features such as cerebellar or brainstem manifestations were also present (3, 5). A participant was classified as pure CA phenotype if the clinical evaluation described CA, either axial or appendicular, with or without abnormal eye movements, and without musculoskeletal symptoms (e.g., stiffness, spasms, and rigidity) (2). Clinical muscle involvement was determined based on distribution of spasms or stiffness on examination. As a clinical disability measure, the modified Rankin Scale (mRS; 0 = no symptoms, 1 = no significant disability, 2 = slight disability, 3 = moderate disability, 4 = moderately severe disability, 5 = severe disability, and 6 = dead) was calculated by a rater blinded to the demographics, clinical presentation, phenotype, and immunological profile of the participants by reviewing only the exams from clinical notes that were closest to the time of the FDG-PET scan (YW).

### FDG-PET Analysis

Two separate group analyses were performed: the first focused on the brain FDG-PET, and the second, on the body FDG-PET.

Dedicated 10-min brain FDG-PET was available in 30 participants and was acquired with filtered back projection (FBP) or RAMP-FBP protocol (24). These scans were analyzed using the NeuroQ software (Syntermed, Atlanta, GA, USA), which provides quantitative analyses of 47 brain region clusters, with comparison with an averaged normal database of 50 individuals without an identified neuropsychiatric or neurodegenerative disease. NeuroQ then provides standardized Z-scores for each brain region cluster (mean of zero and each standard deviation being 1.0), with values above zero indicating hypermetabolism and values below zero indicating hypometabolism. A standard deviation >1.65 was considered significantly hypermetabolic, and <-1.65 was considered significantly hypometabolic, based on recommendations of the NeuroQ manufacturer. Brain MRI was available in all participants, and when multiple scans were available, the scan closest in time to the FDG-PET was reviewed to make sure there were not alternative and/or superimposed findings that could alter the results of the FDG-PET scans.

To review muscle metabolism, an expert nuclear medicine radiologist (LS) reviewed the body FDG-PET studies on a Mirada XD3 workstation (Mirada Medical, Oxford, UK), to determine presence and pattern of abnormal metabolism in muscle. This radiologist was blinded to the demographics, clinical presentation, phenotype, exam findings, and immunological profile of the participants. The results were compared with clinical muscle involvement as determined by review of the clinical note closest in time to the date of the FDG-PET scan (median of 14.5 days, with a range of 0–345 days).

### Statistical Analysis

**Figure 1:**
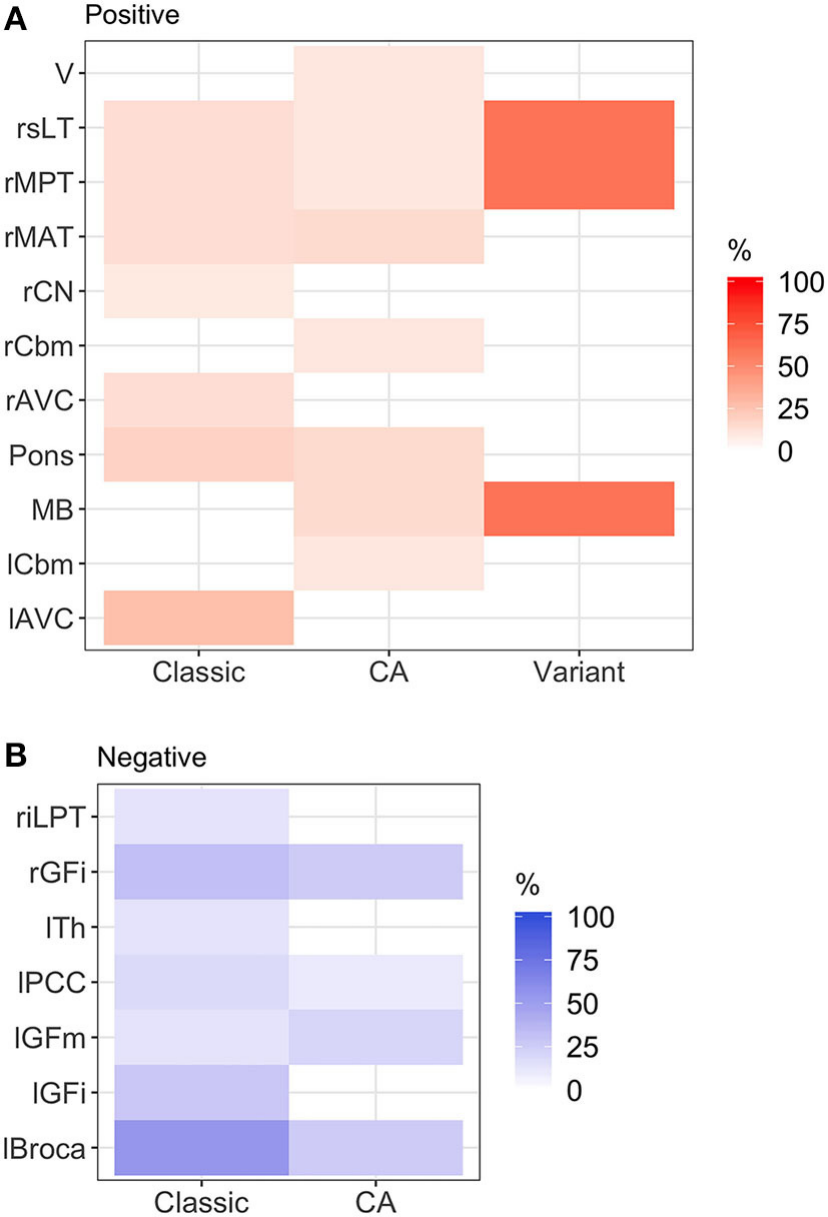
Heatmap of abnormal brain regions by phenotype. **(A)** Hypermetabolic brain regions: brainstem hypermetabolism was seen in all phenotypes. Cerebellar hypermetabolism (left cerebellum, right cerebellum, and vermis) was seen in pure cerebellar ataxia. **(B)** Hypometabolic brain regions: hypometabolism was seen more commonly in classic stiff person syndrome (SPS) phenotype. Classic, classic SPS; CA, pure cerebellar ataxia; Variant, SPS-plus. Abnormal brain region was defined by Z-score >1.65 or <-1.65. rGFi, right inferior frontal cortex; lPCC, left posterior cingulate cortex; lGFi, left inferior frontal cortex; lGFm, left mid frontal cortex; lTh, left thalamus; rMAT, right anterior medial temporal cortex; rCN, right caudate nucleus; MB, midbrain; rMPT, right posterior medial temporal cortex; rsLT, right superior lateral temporal cortex; lAVC, left associative visual cortex; riLPT, right inferior lateral posterior temporal cortex; rCbm, right cerebellum; rMAT, right anterior medial temporal cortex; V, vermis; rMPT, right posterior medial temporal cortex; lCbm, left cerebellum; rsLT, right superior lateral temporal cortex.

Student's *t*-tests were performed to evaluate whether the FDG-PET Z-scores differed from the baseline (zero value). We used heatmaps to illustrate the abnormalities in each group of classic SPS, CA, and SPS-plus, with abnormal values being Z-score >1.65 or < −1.65 as previously stated, with darker colors showing higher percentage of abnormal regions presented (Figure 1). Additionally, we assessed the associations between the mRS and the Z-scores of each brain cluster using multivariate regression models after adjusting for the subjects' phenotype, age, sex, time since symptom onset, and timing of immune therapy (when applicable). Additionally, analyses were performed to evaluate the associations between mRS and the number of abnormal regions, and the number of positive or negative abnormal regions with adjustment for phenotype, age, sex, time since symptom onset, and timing of immune therapy (when applicable). All statistical analyses were performed using R programming (version 3.6.1).

### Data Availability Statement

Anonymized data will be shared by request from qualified investigators.

## Results

### Demographics and Characterization of Study Participants

Demographics, clinical characteristics, and immunological profile of the participants are outlined in Table 1. The clinical profile was similar between the overall cohort and the cohort that included brain FDG-PET. Overall, the average age of symptom onset was 41.5 years (range 12–75 years), majority were female (68%), and majority were White/Caucasian (64%). The main clinical phenotype within the cohort was SPS (41/50, 82%), with 29 of those being classic SPS and 12 being SPS-plus. The remaining participants had pure CA (9/50, 18%). An underlying malignancy was uncovered in three with the FDG-PET screening (two breast cancer and one small cell lung cancer), with use of body imaging including CT scan and FDG-PET. A large proportion had coexisting autoimmune conditions, including diabetes mellitus (insulin-dependent) in 12% and systemic autoimmune disorders in 38% (most commonly autoimmune thyroiditis and pernicious anemia). The majority (47/50) had abnormally elevated serum anti-GAD65 antibody levels, the mean titer level was 65,431 units/ml, and a few individuals had other antibodies identified (two with anti-amphiphysin and one with anti-glycine receptor antibodies), and one had both anti-GAD65 and anti-glycine receptor antibodies. In participants with SPS phenotype whose electromyography (EMG) studies were available for review, 46% had findings that can be seen in SPS (including co-contraction of agonist and antagonist muscles, and continuous muscle fiber/motor unit activity). In a subset of participants who underwent a lumbar puncture, anti-GAD65 antibodies were detected in approximately half, with the range of titers being 0.94–167 nmol/L and median being 18.6 nmol/L. All participants underwent a brain MRI, and in only a minority (three) were abnormalities identified that could alter interpretation of the brain PET scans (one with T2/fluid-attenuated inversion recovery (FLAIR) hyperintense signal in the mesial left temporal lobe, and two with cerebellar volume loss).

**Table 1:** Participant demographics, clinical characteristics, and immunological profile.

**Characteristic**	**Body PET (*n* = 50)**	**Brain PET (*n* = 30)**
Age at onset, years, mean (range)	41.5 (12–75)	40.2 (12–75)
Female, *n* (%)	34 (68)	20 (67)
Race, *n* (%)	White 32 (64) Black 14 (28) Other 4 (8)	White 16 (54) Black 10 (33) Other 4 (13)
**Clinical phenotype**, ***n*****(%)**		
Stiff person syndrome Classic Variant Pure cerebellar ataxia	41 (82) 29 12 9 (18)	22 (73) 16 6 8 (27)
Paraneoplastic, *n*	3^*^	2^**^
Diabetes mellitus, *n* (%)	6 (12)	4 (13)
Other autoimmune disorders, *n* (%) Autoimmune thyroiditis Pernicious anemia Systemic lupus erythematosus Sjogren's syndrome Rheumatoid arthritis Vitiligo Myasthenia gravis Raynaud's phenomenon Mixed connective tissue disorder Psoriasis	19 (38) 9 3 2 2 2 2 1 1 1 1	13 (43) 6 3 2 1 2 1 1 1 0 0
**Serum autoantibody status**, ***n*****(%)**		
Anti-GAD65 alone Anti-amphiphysin Anti-GAD65 and anti-glycine receptor Anti-glycine receptor	46 (92) 2 1 1	29 (97) 0 1 0
Serum anti-GAD65 titer, mean (range), international units/ml	65,431 (0–256,000)^***^	71,831 (7.8–256,000)^****^
**CSF autoimmune profile**		
Anti-GAD abnormal, *n*/total (%) Oligoclonal bands present, *n*/total (%)	18/31 (58) 16/35 (46)	10/20 (50) 9/22 (41)
Abnormal EMG, *n*/total (%)	17/37 (46)	8/21 (38)
Abnormal initial brain MRI, n (%)	3 (6)^*****^	3 (10)^*****^
Modified Rankin Scale, mean (SD)	3.0 (0.92)	3.2 (0.85)

*GAD, glutamic acid decarboxylase; CSF, cerebrospinal fluid; EMG, electromyography*.

* *Two cases of breast cancer and one case of small cell lung cancer*.

** *Two cases of breast cancer*.

*** *Three individuals had a different assay and were excluded from th mean calculation, and their serum anti-GAD65 titers were 0.12, 0.13 and 2483 nmol/L*.

**** *Two individuals had a different assay and were excluded from the mean calculation, and their serum anti-GAD65 titers were 0.12 and 2483 nmol/L*.

***** *Abnormal brain MRI findings were as follows: one with T2/fluid-attenuated inversion recovery (FLAIR) hyperintense signal in the mesial left temporal lobe, and two with cerebellar volume loss*.

### Brain FDG-PET Metabolism Patterns

**Figure 2:**
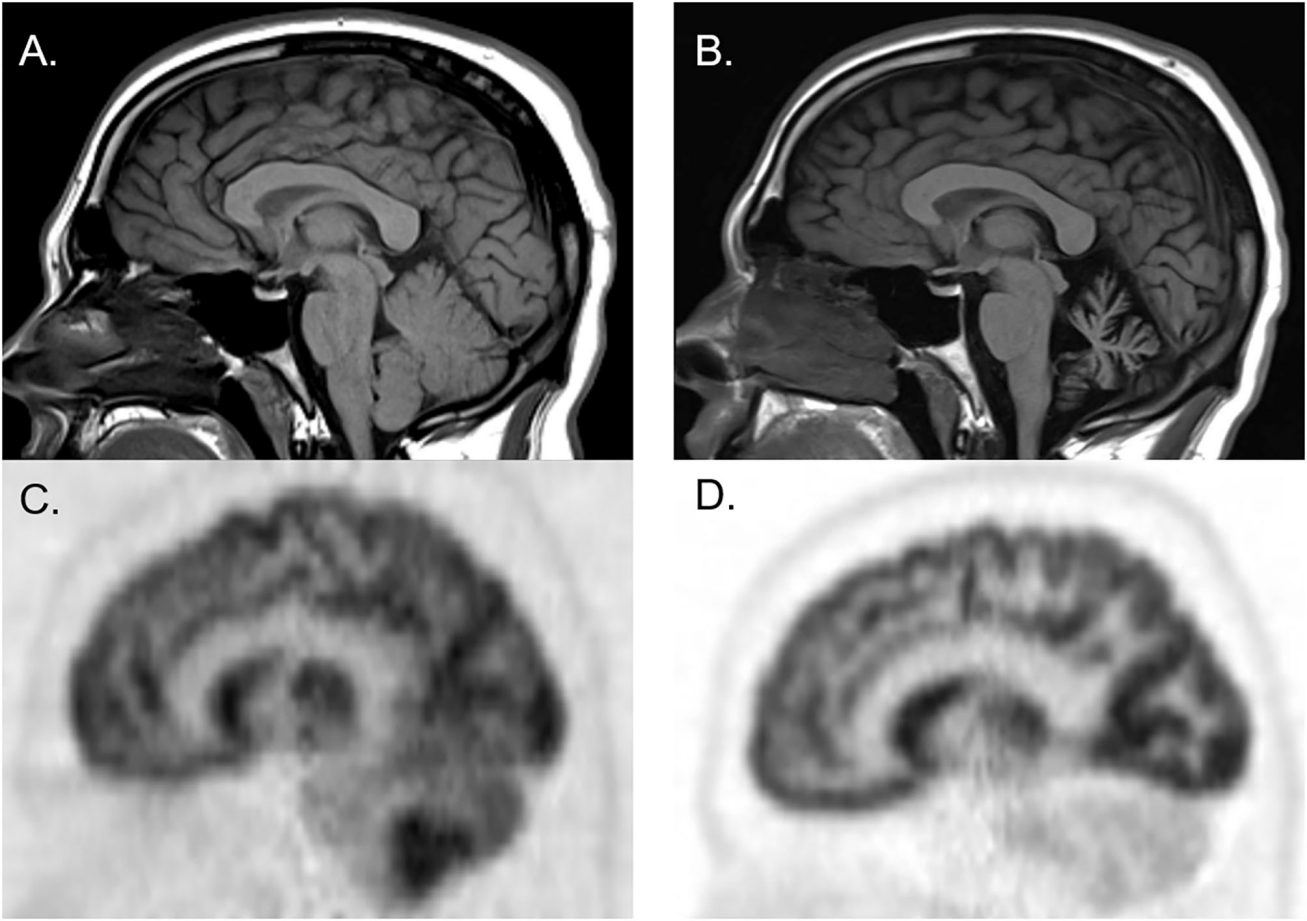
Brain MRI and FDG-PET CT of a man who initially presented with cerebellar ataxia with elevated serum anti-glutamic acid decarboxylase-65 antibodies. At initial presentation, brain MRI was normal [**(A)** sagittal], whereas brain FDG-PET showed hypermetabolism of cerebellar hemispheres [**(C)** sagittal]. Three years later, follow-up brain MRI showed marked cerebellar atrophy [**(B)** sagittal], and brain FDG-PET showed hypometabolism of cerebellar hemispheres [**(D)** sagittal].

Overall, there were various patterns of brain region hypo- and hypermetabolism, as highlighted in Figure 2, which reviewed the frequency of significant hypo- (Figure 2B) or hypermetabolism (Figure 2A) (Z-score ≤−1.65 or ≥ 1.65, respectively), separated by the three phenotypes. Additionally, in Supplementary Table 1, we provide the mean Z-scores of brain regions that were statistically different from control.

In classic SPS, various regions in the frontal, temporal, and occipital lobes demonstrated metabolic abnormalities. The thalamus exhibited hypometabolism, while brainstem regions (midbrain and pons) exhibited hypermetabolism. In pure CA, hypermetabolic changes were more frequently seen, particularly within brainstem and cerebellar regions. In SPS-plus phenotype, metabolic changes were seen in the frontal and temporal lobes, as well as the brainstem, and were most frequently hypermetabolic.

When assessing whether there was any significant correlation between specific brain region *Z*-scores and mRS, only the left inferior lateral posterior temporal cortex (liLPT) showed such correlation (coefficient 0.249, *p* = 0.045). Neither age, nor sex, nor phenotype was associated with mRS.

Figure 1 illustrates the abnormalities in each group of classic SPS, CA, and SPS-plus, with darker colors showing higher percentage of abnormal regions presented (as defined by *Z*-score ≤1.65 or ≥1.65). We noted brainstem hypermetabolism in all phenotypes. Cerebellar hypermetabolism was seen in pure CA, but not SPS phenotype. Figure 2 demonstrates the brain FDG-PET findings of a 34-year-old man who initially presented with CA—though his brain MRI was normal, FDG-PET showed avid update in the bilateral cerebellar hemispheres. Interestingly, he had follow-up FDG-PET performed after 3 years, which showed decreased update in the bilateral cerebellar hemispheres, and at that time, the brain MRI demonstrated marked cerebellar atrophy.

### Muscle FDG-PET Metabolism Patterns

**Figure 3:**
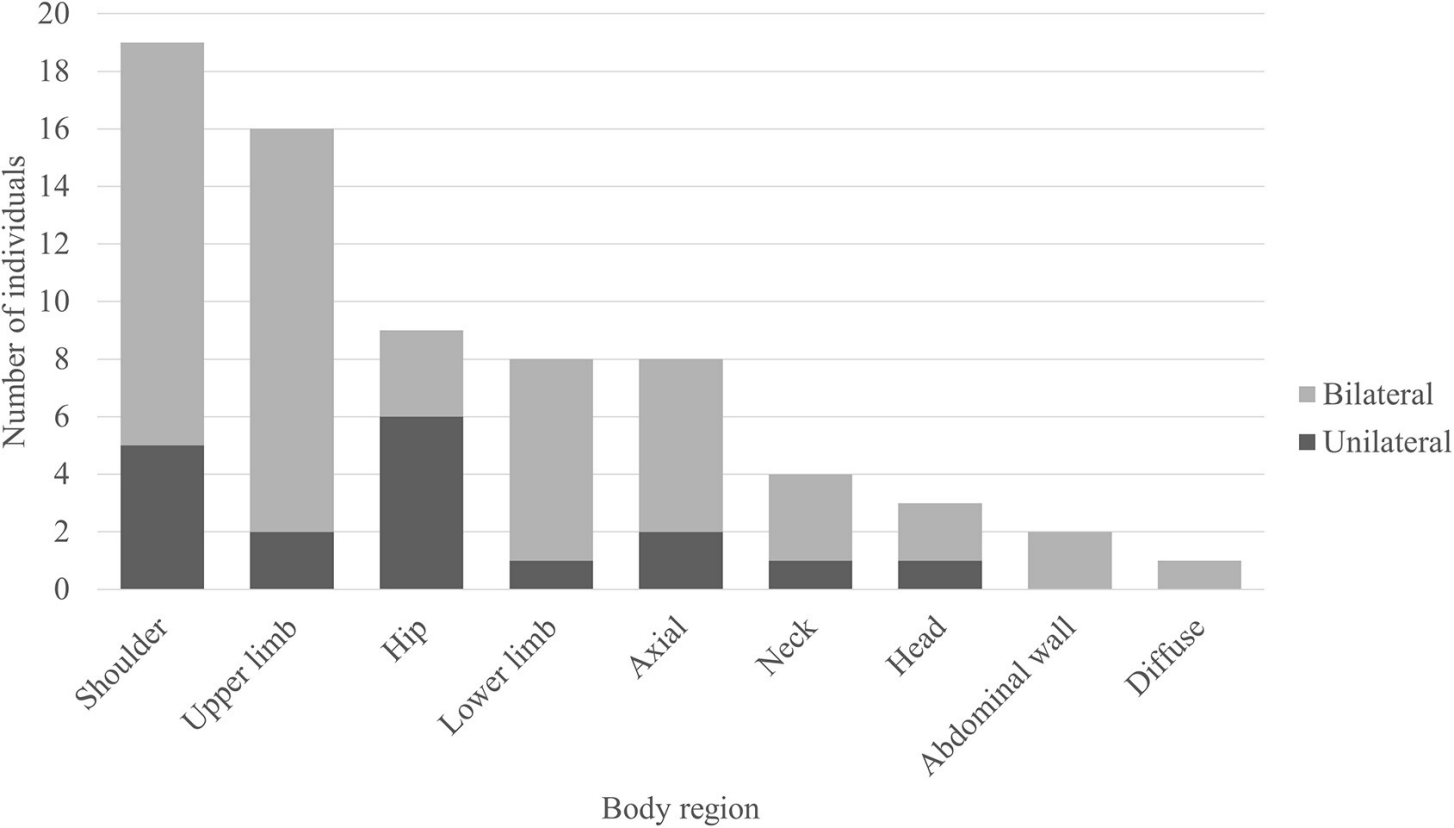
Regions of muscle hypermetabolism on body FDG-PET. Bar graph demonstrating the frequency of regional muscle fluorodeoxyglucose (FDG) avidity in individuals with anti-glutamic acid decarboxylase-65-related neurologic disorder. Shoulder = girdle, infraspinatus, supraspinatus, subscapularis, teres complex. Upper limb = biceps, forearms, deltoids, hands. Hip = iliopsoas, iliacus, psoas. Lower limb = thighs (anterior and posterior compartments), visualized tibialis anterior, gastrocnemius, and soleus. Axial = pectoralis muscles/chest wall, paravertebral muscles (thoracic and/or lumbar regions), gluteal muscles. Neck = paravertebral muscles (cervical region), scalene. Head = occiput. Abdominal wall = obliques, arcus abdominis.

Out of the 50 body FDG-PET reviewed, 31 (62%) had evidence of abnormal muscle hypermetabolism. By phenotype, 26/41 (63%) of those were with SPS phenotype and 5/9 (56%) of those were with pure CA. Figure 3 shows the body region distribution of abnormal muscle hypermetabolism. Shoulders and upper limbs were most commonly hypermetabolic, followed by hips, lower limbs, and axial musculature. Muscle hypermetabolism was most commonly noted to be bilateral. The muscle regions involved correlated with clinical muscle involvement in 42% of individuals. In 62% of participants (21 of 33 available EMG for review), EMG findings correlated with regions of abnormal PET. The caveat was that EMG obviously does not sample all muscles that are visualized on PET. No correlations with clinical outcome (mRS) were found. Supplementary Figure 1 shows an example body FDG-PET scan of a 53-year-old woman with systemic lupus erythematous who presented with 10 years of progressive stiffness and spasms affecting her axial musculature and proximal lower limbs—she had elevated serum anti-GAD65 and abnormal EMG with co-contraction of agonist and antagonist muscles and continuous muscle fiber activity.

## Discussion

This study aimed to characterize brain and muscle metabolic patterns in individuals with SPSSD, by reviewing FDG-PET in both qualitative and quantitative fashion. This study is the largest cohort of such patients with FDG-PET in the literature.

FDG allows for the assessment of glucose metabolism *in vivo* and is the most commonly used tracer for both clinical and research PET-CT imaging. In neurology, FDG-PET is used most commonly for evaluation of neurodegenerative disorders, (25) underlying malignancy, and, more recently, neuroinflammatory disorders (19–21). In individuals with an anti-GAD65-related neurologic disorder, such as SPS or CA, malignancy screening is often performed, given its (albeit rare) association with cancer. Screening for malignancy using FDG-PET is routinely available in clinical practice and has been shown to be cost-effective for screening and staging (26). The addition of dedicated brain acquisitions does not require additional radiotracer administration and is not time-intensive (~10 min).

Currently, there is limited knowledge regarding the full spectrum of metabolic changes in the brain of anti-GAD65-related neurologic disorders. The majority of reported cases in literature with FDG-PET are in individuals presenting with encephalitis, rather than SPS or CA phenotypes. These cases showed mesio-temporal metabolic changes (both hyper- and hypometabolism) in individuals who presented with limbic encephalitis and/or epilepsy, associated with elevated anti-GAD65 (27–32). One case report showed bifrontal hypometabolism in an older woman who presented with cognitive impairment and had the presence of anti-GAD65 in serum and cerebrospinal fluid (CSF) (33). There are also a few case reports of cerebellar degeneration with hypometabolic changes in the cerebellum, correlating with cerebellar atrophy on MRI; the majority of reported cases are associated with anti-Yo (34).

In our cohort of 30 individuals with dedicated brain FDG-PET, we identified various regions of metabolic changes in both SPS and CA phenotypes when compared with individuals without neurodegenerative or neuropsychiatric disorders. Importantly, the regions of involvement did not appear to be solely related to a medication effect since there were asymmetric and region-specific findings that appear to mirror (in part) what is seen clinically, particularly in cases of pure CA. Certain medications commonly used in the treatment of SPS, in particular, benzodiazepines, may produce brain hypometabolic changes in various cortical regions; however, these changes are generally symmetric or global.

The involvement of various cortical regions and thalami in SPS is not surprising—GABAergic neurons are present throughout the brain, and the thalami are important components of connecting the cortex to the striatal network, which are affected in various movement disorders. Interestingly, brainstem regions were involved in all phenotypes; though more commonly seen in the CA phenotype, the involvement of these regions in other phenotypes may further support the thought that these conditions lie in a spectrum, from pure CA to classic SPS. (10) This finding was a bit unexpected, as SPS is not felt to have significant brainstem dysfunction, though there have been limited previous studies suggesting hyperexcitability of brainstem interneural circuits in SPS; thus, this region may deserve further study in SPS (35). What is unknown and needs to be further elucidated is the susceptibility of certain brain regions to impaired neuronal activity and how that affects the clinical presentation, though there certainly seem to be hints at this based on our results, particularly in individuals with pure CA, who showed metabolic changes in the brainstem and cerebellum. There are also autopsy studies in patients with CA and anti-GAD65 showing selective loss of Purkinje cells (36, 37). It is possible that in anti-GAD65-related neurologic disorders, certain brain regions may be preferentially affected, producing distinctive phenotypes with specific signs and symptoms.

Currently, the pathological mechanisms underlying anti-GAD65-related neurologic disorders, and their varied presentations, are largely unknown. GAD is an enzyme that catalyzes the conversion of glutamate to GABA, and within the CNS, it is expressed in inhibitory neurons, which are ubiquitously present, though there may be regional differences. Recent studies have shown that these GABAergic inhibitory neurons have important functions as part of the cerebrocerebellar loop, connecting the motor cortex to the cerebellum (38, 39).

Though FDG-PET metabolic changes are not specific, the technology may aide in elucidating the underlying pathophysiology of metabolic changes. For example, in neurodegenerative disorders such as Alzheimer's dementia and amyotrophic lateral sclerosis, it is hypothesized that hypometabolism represents neurodegeneration and axonal loss. On the other hand, in neuroinflammation and glial activation, hypermetabolic changes may be seen (40). Both neuroinflammation and neurodegeneration may underlie anti-GAD65-related neurologic disorders. Our results show that metabolic patterns in brain regions affected differ based on disease phenotype. An additional interesting observation from one of the patients (Figure 3) may point toward differing stages of the disease—this individual exhibited selective cerebellar hypermetabolism early in the disease course, followed by neuronal loss. Though further work is necessary to elucidate the mechanism underlying such changes, they may be helpful in diagnosis, and also potentially to identify those who may respond to immune therapy, which mostly target the inflammatory phase. This would be particularly important because patients may improve or halt their clinical decline with early use of immunotherapy (41).

In a review of the muscle metabolic changes, the majority of individuals demonstrated abnormal FDG uptake. Though we did not have a control group for comparison, previous studies have demonstrated that incidentally noted abnormal muscle FDG uptake may be seen in about 12.5% of individuals who presented with no musculoskeletal concerns (42). As expected, based on phenotype, lower limbs and axial muscles were affected, but surprisingly, we also saw a large percentage with upper limb involvement. Though the correlation between clinical muscle involvement (with stiffness and/or spasms) and presence of FDG uptake was <50%, there were likely multiple contributors to this, including time interval between examination and the FDG-PET, as well as symptomatic treatments (including muscle relaxants) that individuals were taking. Interestingly, we also found abnormal muscle FDG uptake in individuals with CA phenotype. Potential mechanisms for this include muscle metabolic changes that may occur as a result of long-term change in posture and/or gait, as well as presence of stiffness and/or spasms that were not captured clinically at the time of the scan, as part of the spectrum of disorders. In the literature, to the best of our knowledge, there is only one case report that reported increased FDG uptake in axial and proximal lower limbs in an individual with SPS (43).

There are some limitations to this study. The FDG-PET scans were not always acquired at the onset of the disease, and it is possible that metabolic changes may differ depending on the stage of the disease and response to various treatments (including immunotherapies). Also, the majority of clinical exams were not performed on the same day as the FDG-PET scan. FDG-PET is still a newer utilized imaging method, particularly in the assessment of autoimmune disease, and FDG is a nonspecific radiotracer, as discussed above. There are other nuclear medicine techniques that may be useful in evaluating anti-GAD65-associated neurologic disorders, specifically those that assess GABAergic function. However, these are not widely available and not the radiotracer used for FDG-PET performed for malignancy screening; thus, this requires specialized compounding and additional scanning. Another limitation is that our control group was an averaged database based on the NeuroQ software, rather than specifically age-matched controls; however, based on previous studies, age-related changes on FDG PET have mostly been noted in the cortices, specifically the frontal cortices, and are not known to significantly affect the brainstem nor cerebellum (44, 45). The study was retrospective and cross-sectional and, as part of its retrospective nature, does have selection bias in terms of individuals in which FDG-PET scans were obtained. In addition, currently, there is a lack of robust outcome measures, and the mRS that was utilized as a clinical outcome measure may not be adequately capturing clinical function in these patients. Further prospective studies would be beneficial to determine metabolic changes in a longitudinal fashion, as this may inform both clinical course and treatment response.

In conclusion, we demonstrate that brain metabolic changes are present in SPSSD (excluding those with refractory epilepsy and encephalitis) and that the regional patterns seen differ based on the clinical phenotype. We also show that muscle hypermetabolic activity is seen in these disorders and in patterns that are unique. FDG-PET is becoming increasingly available and utilized around the world. It is often used for the identification of occult malignancy in patients with suspected paraneoplastic syndrome. Future prospective studies would better inform the potential complementary role of FDG-PET in the diagnosis and monitoring of individuals with SPSSD and will potentially allow us to better understand the expanding spectrum of anti-GAD65-associated neurologic disorders.

## Data Availability Statement

The raw data supporting the conclusions of this article will be made available by the authors, without undue reservation.

## Ethics Statement

The studies involving human participants were reviewed and approved by Johns Hopkins University IRB. The patients/participants provided their written informed consent to participate in this study.

## Author Contributions

SN, LS, and MS contributed to conception and design of the study. KF performed the statistical analysis. FT performed the statistical analysis, wrote sections of the manuscript. YW contributed to conception and design of the study, wrote the first draft of the manuscript. All authors contributed to manuscript revision, read, and approved the submitted version.

## Conflict of Interest

LS reports being a principal investigator and has received salary support from Progenics Azedra trial. SN has received consultant fees for scientific advisory boards from Biogen, Genentech, Celgene, EMD Serrono, and Novartis; is an advisor for Autobahn Therapeutics and BioIncept and a clinical adjudication committee member for a medDay Pharmaceuticals clinical trial; and has received research funding (paid directly to institution) from Biogen, Novartis, Genentech, National Multiple Sclerosis Society, Department of Defense, and Patient-Centered Outcomes Research Institute. The remaining authors declare that the research was conducted in the absence of any commercial or financial relationships that could be construed as a potential conflict of interest.

## Publisher's Note

All claims expressed in this article are solely those of the authors and do not necessarily represent those of their affiliated organizations, or those of the publisher, the editors and the reviewers. Any product that may be evaluated in this article, or claim that may be made by its manufacturer, is not guaranteed or endorsed by the publisher.

## Supplementary Material

Supplementary Figure 1 Body FDG-PET CT of a middle-aged woman with systemic lupus erythematosus. She presented with ten years of progressive axial and lower extremity stiffness and spasms. Her serum level of anti-glutamic acid decarboxylate 65 antibody was elevated, and electromyography showed co-contraction of agonist and antagonist muscles and continuous muscle fiber activity in the anterior tibial and medial gastrocnemius muscles. FDG-PET CT scan showed increased FDG uptake in her posterior neck muscles as well as quadriceps and iliacus bilaterally, in coronal **(A)** and axial **(B)** sections.

Supplementary Table 1 Z-scores of brain region metabolic activity measured in FDG-PET scans of patients stiff person syndrome spectrum disorders.
