# Supplementary figures and images for: Brain and Muscle Metabolic Changes by FDG-PET in Stiff Person Syndrome Spectrum Disorders
Source: Front Neurol. 2021 Sep 17;12:692240. doi: 10.3389/fneur.2021.692240 (PMC8484315; doi:10.3389/fneur.2021.692240)

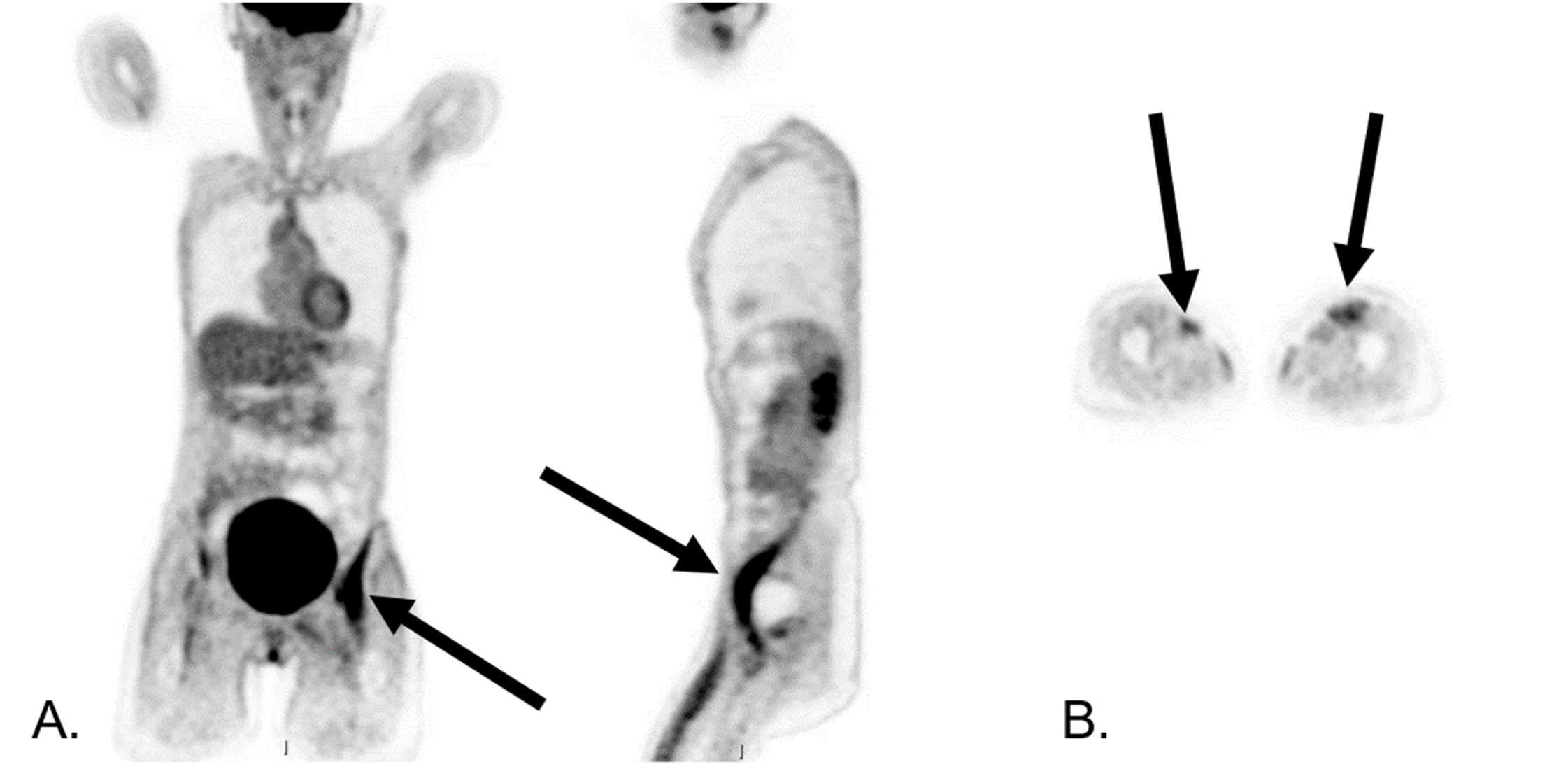

Supplement: Supplementary Figure 1 — Body FDG-PET CT of a middle-aged woman with systemic lupus erythematosus. She presented with ten years of progressive axial and lower extremity stiffness and spasms. Her serum level of anti-glutamic acid decarboxylate 65 antibody was elevated, and electromyography showed co-contraction of agonist and antagonist muscles and continuous muscle fiber activity in the anterior tibial and medial gastrocnemius muscles. FDG-PET CT scan showed increased FDG uptake in her posterior neck muscles as well as quadriceps and iliacus bilaterally, in coronal (A) and axial (B) sections. [file Image_1.TIF]
