# Supplementary material for: Brain and Muscle Metabolic Changes by FDG-PET in Stiff Person Syndrome Spectrum Disorders
Source: Front Neurol. 2021 Sep 17;12:692240. doi: 10.3389/fneur.2021.692240 (PMC8484315; doi:10.3389/fneur.2021.692240)
Supplement: Supplementary Table 1 — Z-scores of brain region metabolic activity measured in FDG-PET scans of patients stiff person syndrome spectrum disorders. [file Table_1.docx]

**Supplemental Table 1:** Z-scores of brain region metabolic activity measured in FDG-PET scans of patients Stiff Person Syndrome Spectrum Disorders

|  | Classic SPS phenotype (n = 16) | SPS-plus phenotype (n = 6) | Pure cerebellar ataxia phenotype (n = 8) |
| --- | --- | --- | --- |
| lBroca, mean (CI) | -2.14 (-2.57 to -1.71) |  | -2.66 (-4.29 to -1.03) |
| rGFi | -1.57 (-2.17 to -0.98) | -1.27 (-2.12 to -0.42) | -1.73 (-2.47 to -0.99) |
| lGFi | -1.09 (-1.59 to -0.59) |  | -0.88 (-1.72 to -0.04) |
| rGFm | -0.619 (-0.98 to -0.26) |  |  |
| lGFm | -1.09 (-1.54 to -0.64) |  | -1.57 (-2.98 to -0.17) |
| lPCC | -1.06 (-1.51 to -0.61) |  | -1.16 (-2.13 to -0.20) |
| rTh |  |  | 0.70 (0.14 to 1.25) |
| lTh | -0.77 (-1.17 to -0.37) |  |  |
| rGFs |  | 0.52 (0.11 to 0.92) |  |
| liLAT | -0.61 (-0.93 to -0.29) |  |  |
| riLPT | -0.84 (-1.25 to -0.43) |  | -0.71 (-1.38 to -0.05) |
| liLPT | -0.44 (-0.83 to -0.05) |  |  |
| rAVC | 0.86 (0.11 to 1.6) |  |  |
| lAVC | 1.222 (0.13 to 2.31) |  |  |
| lSM |  |  | -0.45 (-0.87 to -0.04) |
| rsLT | 1.15 (0.75 to 1.55) | 2.18 (0.33 to 4.03) | 1.15 (0.05 to 2.25) |
| lsLT | 0.64 (0.22 to 1.06) |  |  |
| rMAT | 0.67 (0.02 to 1.31) |  | 1.04 (0.06 to 2.02) |
| lMAT |  |  | 1.08 (0.49 to 1.67) |
| rMPT | 0.63 (0.13 to 1.12) | 1.99 (0.07 to 3.91) | 1.43 (0.36 to 2.51) |
| lMPT |  |  | 0.69 (0.11 to 1.27) |
| lsPL | 0.42 (0.04 to 0.79) |  |  |
| rCN | 0.72 (0.39 to 1.05) |  | 0.70 (0.15 to 1.25) |
| lCN | 0.41 (0.02 to 0.8) |  |  |
| rLN | 0.33 (0.09 to 0.58) |  |  |
| lLN |  |  | 0.63 (0.03 to 1.22) |
| pons | 1.06 (0.19 to 1.93) |  | 1.67 (0.59 to 2.74) |
| MB | 0.47 (0.01 to 0.93) | 1.36 (0.24 to 2.48) | 1.26 (0.55 to 1.97) |
| V |  |  | 1.07 (0.22 to 1.92) |
| rCbm |  |  | 0.79 (0.03 to 1.55) |
| lCbm |  |  | 0.99 (0.08 to 1.89) |

CI = confidence interval. lBroca = left Broca’s region; rGFi = right inferior frontal ccortex; lPCC = left posterior cingulate cortex; rLPT = right inferior lateral posterior temporal cortex; lGFi = left inferior frontal cortex; liLPT = left inferior lateral posterior temporal cortex; lGFm = left mid frontal cortex; liLAT = left inferior lateral anterior temporal cortex; rGFm = right mid frontal cortex; lTh = left thalamus; lsPL = left superior parietal cortex; rLN = right lentiform nucleus; rMAT = right anterior medial temporal cortex; lCN = left caudate nucleus; rCN = right caudate nucleus; lsLT = left superior lateral temporal cortex; MB = midbrain; rAVC = right associative visual cortex; rMPT = right posterior medial temporal cortex; rsLT = right superior lateral temporal cortex; lAVC = left associative visual cortex; riLPT = right inferior lateral posterior temporal cortex; lSM = left sensorimotor cortex; lLN = left lentiform nucleus; rCbm = right cerebellum; lMPT = left posterior medial temporal cortex; rTh = right thalamus; rMAT = right anterior medial temporal cortex; V = vermis; rMPT = right posterior medial temporal cortex; lCbm = left cerebellum; rsLT = right superior lateral temporal cortex; lMAT = left anterior medial temporal cortex
